# The arylpiperazine derivatives *N*-(4-cyanophenylmethyl)-4-(2-diphenyl)-1-piperazinehexanamide and *N*-benzyl-4-(2-diphenyl)-1-piperazinehexanamide exert a long-lasting inhibition of human serotonin 5-HT_7_ receptor binding and cAMP signaling

**DOI:** 10.1002/prp2.13

**Published:** 2013-12-05

**Authors:** Patricio Atanes, Enza Lacivita, Javier Rodríguez, José Brea, Javier Burgueño, José Miguel Vela, María Isabel Cadavid, María Isabel Loza, Marcello Leopoldo, Marián Castro

**Affiliations:** 1 Biofarma Research Group, Department of Pharmacology, Center for Research in Molecular Medicine and Chronic Diseases (CIMUS), University of Santiago de CompostelaSantiago de Compostela, Spain; 2 Dipartimento di Farmacia – Scienze del Farmaco, Università degli Studi di Bari “A. Moro”Bari, Italy; 3 Esteve; Drug Discovery and Preclinical DevelopmentBarcelona, Spain

**Keywords:** [^3^H]-SB-269970 binding, arylpiperazine derivative, cAMP signaling, HEK293 cells, insurmountable antagonism, irreversible inhibition, long-lasting inhibition, LP-211, preincubation/washout experiments, Schild analysis, serotonin 5-HT_7_ receptors

## Abstract

We performed a detailed in vitro pharmacological characterization of two arylpiperazine derivatives, compound *N*-(4-cyanophenylmethyl)-4-(2-diphenyl)-1-piperazinehexanamide (LP-211) previously identified as a high-affinity brain penetrant ligand for 5-hydroxytryptamine (serotonin) type 7 (5-HT_7_) receptors, and its analog *N*-benzyl-4-(2-diphenyl)-1-piperazinehexanamide (MEL-9). Both ligands exhibited competitive displacement of [^3^H]-(2*R*)-1-[(3-hydroxyphenyl)sulfonyl]-2-[2-(4-methyl-1-piperidinyl)ethyl]pyrrolidine ([^3^H]-SB-269970) radioligand binding and insurmountable antagonism of 5-carboxamidotryptamine (5-CT)-stimulated cyclic adenosine monophosphate (cAMP) signaling in human embryonic kidney (HEK293) cells stably expressing human 5-HT_7_ receptors. They also inhibited forskolin-stimulated adenylate cyclase activity in 5-HT_7_-expressing HEK293 cells but not in the parental cell line. The compounds elicited long-lasting (at least 24 h) concentration-dependent inhibition of radioligand binding at 5-HT_7_-binding sites in whole-cell radioligand binding assays, after pretreatment of the cells with the compounds and subsequent compound removal. In cAMP assays, pretreatment of cells with the compounds rendered 5-HT_7_ receptors unresponsive to 5-CT and also rendered 5-HT_7_-expressing HEK293 cells unresponsive to forskolin. Compound 1-(2-biphenyl)piperazine (RA-7), a known active metabolite of LP-211 present in vivo, was able to partially inhibit 5-HT_7_ radioligand binding in a long-lasting irreversible manner. Hence, LP-211 and MEL-9 were identified as high-affinity long-acting inhibitors of human 5-HT_7_ receptor binding and function in cell lines. The detailed in vitro characterization of these two pharmacological tools targeting 5-HT_7_ receptors may benefit the study of 5-HT_7_ receptor function and it may lead to the development of novel selective pharmacological tools with defined functional properties at 5-HT_7_ receptors.

## Introduction

The serotonin 5-hydroxytryptamine (serotonin) type 7 (5-HT_7_) receptor is a G_s_-coupled receptor (Hoyer et al. 2002) expressed in discrete areas of the brain (particularly in hippocampus, thalamus, and hypothalamus) and periphery (Jasper et al. 1997; Regard et al. 2008). Its distribution correlates well with the functions attributed to the receptor in learning/memory, regulation of mood, thermoregulation, nociception or circadian rhythms, among others (Hedlund and Sutcliffe 2004; Leopoldo et al. 2011). The interest in 5-HT_7_ receptors as drug target in clinical conditions such as depression, schizophrenia, sleep disorders, migraine, or neuropathic pain (Hedlund 2009; Stahl 2010; Matthys et al. 2011; Meltzer and Massey 2011; Bonaventure et al. 2012; Henigsberg et al. 2012; Cates et al. 2013), together with current efforts aimed to develop the first 5-HT_7_-selective positron emission tomography radioligand (Lemoine et al. 2011), warrants investigations of the mechanisms of action of compounds acting at these receptors.

Progress in 5-HT_7_ research has been hampered by the lack of selective ligands. However, the recent development and characterization of selective compounds (Pittalà et al. 2007; Brenchat et al. 2009; Leopoldo et al. 2011), combined with molecular biology techniques, is helping unveil the rich 5-HT_7_ pharmacology. We have previously developed different series of 4-substituted 1-arylpiperazine derivatives (Perrone et al. 2003; Leopoldo et al. 2004a,b, 2007, 2008). Among these, compound *N*-(4-cyanophenylmethyl)-4-(2-diphenyl)-1-piperazinehexanamide (LP-211) exhibited nanomolar affinity for rat (*K*_i_ = 0.58 nmol/L) and human (*K*_i_ = 15 nmol/L) 5-HT_7_ receptors cloned in cell lines (Leopoldo et al. 2008; Hedlund et al. 2010), and lower affinities for 5-HT_1A_ (*K*_i_ = 188–379 nmol/L) (Leopoldo et al. 2008; Hedlund et al. 2010), D_2_ (*K*_i_ = 142 nmol/L) (Leopoldo et al. 2008), and for all other human serotonin receptor subtypes and the human serotonin transporter (SERT) (Hedlund et al. 2010). This compound was able to modulate the capsaicin-induced increase in FBJ murine osteosarcoma viral oncogene homolog (Fos)-like immunoreactivity, a marker of cephalic pain, in rat trigeminal nucleus caudalis (Martínez-García et al. 2011). LP-211 has been proposed as a tool for rescuing the fragile X syndrome phenotype in the Fmr1 (fragile X mental retardation 1) knockout mice model (Costa et al. 2012). It has also been postulated as a suitable tool for studying the role of 5-HT_7_ receptor systems in sleep disorders and in the modulation of emotional and motivational limbic systems (Adriani et al. 2012). In the case of compound *N*-benzyl-4-(2-diphenyl)-1-piperazinehexanamide (MEL-9), a close structural analog of LP-211, its *K*_i_ value at human 5-HT_7_ receptors was estimated to be 0.8 nmol/L from experiments in which the compound showed a behavior not compatible with classical competitive binding (Leopoldo et al. 2008). MEL-9 displayed lower affinities for 5-HT_1A_ and D_2_ receptors (*K*_i_ = 503 nmol/L and 161 nmol/L, respectively) (Leopoldo et al. 2008). In spite of their interest as selective 5-HT_7_ ligands and pharmacological tools, neither of these two compounds has been functionally characterized in detail in terms of 5-HT_7_ down-stream signaling in well-controlled in vitro settings such as a cell line.

The development of drugs and novel chemical tools selective and active at 5-HT_7_ receptors has important implications for 5-HT_7_ receptor research and central nervous system pharmacology and therapy, which justifies the interest in detailed descriptions of their underlying mechanisms of action. Therefore, in this study, we aimed to undertake a detailed pharmacological characterization of the mechanism of action of compound LP-211 and its analog MEL-9, at human 5-HT_7_ receptors expressed in human embryonic kidney (HEK293) cells. The results indicate that both compounds behave as long-lasting inhibitors of 5-HT_7_ receptors. These ligands may lead to the development of novel selective pharmacological tools with defined functional properties at 5-HT_7_ receptors.

## Materials and Methods

### Reagents

Compounds MEL-9, LP-211, and 1-(2-biphenyl)piperazine (RA-7) were synthesized at the Dipartimento di Farmacia – Scienze del Farmaco, Università degli Studi di Bari “A. Moro” as previously described (Leopoldo et al. 2008; Lacivita et al. 2012). Clozapine, methiothepin mesylate salt (methiothepin), 5-carboxamidotryptamine maleate salt (5-CT), and forskolin were purchased from Sigma-Aldrich Quimica SL (Madrid, Spain). [^3^H]-SB-269970 ((2*R*)-1-[(3-hydroxyphenyl)sulfonyl]-2-[2-(4-methyl-1-piperidinyl)ethyl]pyrrolidine) (39.9 Ci/mmol) was purchased from PerkinElmer (Madrid, Spain). All other reagents were purchased from Sigma-Aldrich Quimica SL (Madrid, Spain), unless otherwise specified.

### Cell culture

HEK293 cells stably expressing the human 5-HT_7(a)_ receptor (HEK-hu5-HT_7_) (at a receptor density of 22 pmol/mg protein) were kindly provided by Laboratorios Esteve (Barcelona, Spain) and have been employed in previous studies (Varin et al. 2010). Cells were grown in culture medium (Dulbecco's Modified Eagle's Medium [4.5 g/l glucose]) supplemented with 10% (v/v) fetal bovine serum, 100 U/mL penicillin, 100 mg/mL streptomycin, GlutaMAX™-I (Gibco, Life Technologies S.A., Madrid, Spain), 2 mM L-glutamine, 1 mM sodium pyruvate, and 0.5 mg/ml geneticin (G418 sulfate, Gibco), at 37°C in a 5% CO_2_ humidified atmosphere.

### Membrane homogenate radioligand binding

Cell membrane homogenate was prepared as previously described (Varin et al. 2010). Membrane homogenate (5 μg protein/tube) was incubated for 60 min at 37°C in 500 μL of binding buffer (50 mmol/L Tris-HCl pH 7.4, 4 mmol/L MgCl_2_, 1 mmol/L ascorbic acid and 0.1 mmol/L pargyline) containing [^3^H]-SB-269970 as radioligand. Nonspecific binding was determined in the presence of 25 μmol/L clozapine. 5-HT_7_ receptor density (*B*_max_ = 21.80 ± 0.76 pmol/mg prot.) and affinity (*K*_d_) of the radioligand for the receptors were determined in saturation binding experiments with concentrations of the radioligand ranging from 0.03 to 30 nmol/L. The affinity (*K*_i_) of MEL-9, LP-211 and methiothepin was determined in competition experiments with 2 nmol/L [^3^H]-SB-269970 in the absence (control) or presence of increasing concentrations (ranging from 1 pmol/L to 10 μmol/L) of the unlabeled compounds. After incubation, the reactions were stopped by filtration in a harvester apparatus through GF/B filters pretreated with 0.25% (w/v) poly(ethyleneimine) solution. Filters were counted in a LS-6000LL beta counter (Beckman Coulter, Inc., Fullerton, CA, USA).

### Whole-cell radioligand binding (assays in the presence of the compounds)

Cells growing in 100-mm culture dishes were detached by addition of 1 mL/plate of a 1:3 dilution of trypsin-ethylenediaminetetraacetic acid (EDTA) solution (0.5 g/L porcine trypsin, 0.2 g/L EDTA•4Na in Hank's Balanced Salt Solution) in phosphate-buffered saline (PBS), and the cells were harvested in 4-(2-hydroxyethyl)-1-piperazineethanesulfonic acid (HEPES) buffer (20 mmol/L HEPES pH 7.5, 2.5 mmol/L MgSO_4_, 134 mmol/L NaCl, 10 μmol/L pargyline) prewarmed at 37°C. The cells were centrifuged at 330*g* for 6 min, and the cell pellet was resuspended in HEPES buffer. 5-HT_7_ receptor density (*B*_max_ = 152.50 ± 5.66 fmol/120,000 cells) and affinity (*K*_d_) of the radioligand for the receptors in whole cells (120,000 cells/tube) were determined in saturation binding experiments with concentrations of the radioligand ranging from 0.03 to 30 nmol/L. The effect of the compounds on [^3^H]-SB-269970 binding to intact cells was determined in competition experiments by incubating 50,000 cells in assay tubes containing 2 nmol/L radioligand in 500 μL HEPES buffer, in the absence (control) or presence of increasing concentrations (ranging from 100 pmol/L to 10 μmol/L) of MEL-9 or LP-211, or of a single concentration (10 μmol/L) of RA-7. Nonspecific binding was determined in the presence of 25 μmol/L clozapine. After incubation for 30 min at 37°C, the assay was stopped, as previously described for membrane homogenate radioligand binding assays.

### Whole-cell radioligand binding (preincubation/washout experiments)

To determine the effects of the compounds on [^3^H]-SB-269970 binding following removal of the compounds, intact cells were detached and harvested, as described for whole-cell radioligand binding assays in the presence of the compounds, and then preincubated in HEPES buffer (10 × 10^6^ cells/mL) in the absence (control) or presence of the compounds under study, at the concentrations indicated for 30 min at 37°C under gentle agitation. The cell suspension was centrifuged at 330*g* for 6 min, the cell pellet was washed three times for 10 min in HEPES buffer at 37°C, and the cells were immediately resuspended in HEPES buffer for [^3^H]-SB-269970 binding assays. For this purpose, 50,000 cells/tube were incubated in assay tubes containing 2 nmol/L [^3^H]-SB-269970 in 500 μL HEPES buffer in the absence or presence of 25 μmol/L clozapine to determine the total and nonspecific binding, respectively. After incubation for 30 min at 37°C, the assay was stopped, as previously described. To determine the time course of recovery of the specific binding of [^3^H]-SB-269970 after removal of the compounds, cells handled under sterile conditions were incubated with the compounds, as indicated above. After compound washout, cells were incubated for different times (*t* = 0, 1, 3, 6, and 24 h) at 37°C in Dulbecco's Modified Eagle Medium:Nutrient Mixture F-12-GlutaMAX™ (Gibco) supplemented with 100 U/mL penicillin and 100 mg/mL streptomycin, under sterile conditions, prior to being subjected to binding assays to determine the total and nonspecific [^3^H]-SB-269970 binding, as described above.

### cAMP assays (assays in the presence of the compounds)

cAMP levels were quantified using the homogeneous time-resolved fluorescence (HTRF)-based cAMP dynamic kit (Cisbio, Bioassays, Codolet, France). Twenty-four hours before the assay, HEK-hu5-HT_7_ cells were plated at a density of 8000 cells/well in Opti-MEM medium (Invitrogen, Life Technologies S.A.) in white polystyrene, tissue-culture treated, half-area Corning 96 well plates pretreated with poly-d-lysine. To measure agonist effects, cells were incubated in cAMP assay buffer (Opti-MEM, 500 μmol/L 3-isobutyl-1-methylxanthine [IBMX]) for 15 min at 37°C prior to the addition of the compounds and further incubation for 15 min. The antagonist effect of the compounds was evaluated in the presence of 5-CT (at the indicated concentration) by constructing concentration–response curves. Cells were incubated in the absence (control) or presence of increasing concentrations (from 100 pmol/L to 10 μmol/L) of the compounds in assay buffer for 15 min at 37°C prior to the addition of the agonist and further incubation for 15 min. For Schild analysis, concentration–response curves were constructed for 5-CT in the absence (control) or presence of the compounds at the concentrations indicated. Cells were incubated with the compounds in assay buffer for 15 min at 37°C prior to addition of increasing concentrations (from 100 pmol/L to 10 μmol/L) of 5-CT and further incubation for 15 min. In all cases, basal cAMP levels were determined in control wells in the absence of agonist. The effects of the compounds on forskolin-stimulated adenylate cyclase activity were evaluated either at a single concentration (parental HEK293 cells) or by using concentration–response curves (HEK-hu5-HT_7_ cells). Cells were incubated in the absence (control) or presence of the compounds, at the concentrations indicated, in assay buffer for 15 min at 37°C prior to the addition of forskolin and further incubation for 15 min. Basal cAMP levels were determined in control wells in the absence of forskolin. After proceeding with the subsequent assay steps according to the manufacturer's protocol, the fluorescence emission intensity ratio at 665/620 nm wavelength was measured in an Ultra Evolution 384 microplate reader (TECAN, Männedorf, Switzerland).

### cAMP assays (preincubation/washout experiments)

To determine the effects of the compounds on 5-CT- or forskolin-stimulated cAMP levels following removal of the compounds, cells plated as previously described for cAMP assays 6 were preincubated in the absence (control) or presence of increasing concentrations (from 100 pmol/L to 10 μmol/L) of the compounds in cAMP assay buffer for 30 min at 37°C. The cells were then washed three times for 10 min in Opti-MEM at 37°C and incubated in cAMP assay buffer with 10 μmol/L 5-CT for 30 min at 37°C, or with 10 μmol/L forskolin for 15 min at 37°C. Basal cAMP levels were determined in control wells in the absence of 5-CT or forskolin in each case. After proceeding with the subsequent assay steps according to the manufacturer's protocol, fluorescence was measured as previously described.

### Data analysis

The data were analyzed using GraphPad Prism software v4.0 (GraphPad Software Inc., San Diego, CA). Receptor density (*B*_max_) and the dissociation constant (*K*_d_) of the radioligand were determined by nonlinear regression curve fitting of saturation binding curves to a one-site binding (hyperbola) model. The inhibitory dissociation constants (*K*_i_) for the compounds under study were calculated using competition binding experiments, according to the Cheng and Prusoff equation: *K*_i_ = IC_50_/(1 + [L]/*K*_d_), where IC_50_ is the concentration of the unlabelled compounds leading to half-maximal inhibition of the specific binding, [L] is the concentration of the radioligand used in the assay and *K*_d_ is the dissociation constant of the radioligand determined by nonlinear regression curve fitting of saturation binding curves to a one-site binding (hyperbola) model (*K*_d_ = 1.53 ± 0.16 nmol/L in membrane homogenate assays; *K*_d_ = 1.49 ± 0.18 nmol/L in whole-cell assays). The IC_50_ values were determined by nonlinear regression curve fitting to sigmoidal dose–response or sigmoidal dose–response (variable slope) models, as indicated. Specific binding was expressed as the percent of specific [^3^H]-SB-269970 binding in control conditions. 5-CT potency (EC_50_) and efficacy (maximal response [*E*_max_]) and antagonist potency (IC_50_) of the compounds in cAMP assays were calculated by fitting the data to a sigmoidal dose–response model. IC_50_ denotes the concentration of compound that causes half-maximal inhibition of cAMP production stimulated by 5-CT or forskolin, and IC_50, irrev_ denotes the potency of the compounds as irreversible inhibitors of 5-HT_7_-mediated cAMP signaling or whole-cell 5-HT_7_ radioligand binding in preincubation/washout experiments. Results are expressed as the mean values ± SEM from the number of independent experiments indicated. Prediction of log of octanol/water partition coefficient (*x*log *P*) values were retrieved from ZINC database (http://zinc.docking.org/) (Irwin et al. 2012).

## Results

**Figure 1:**
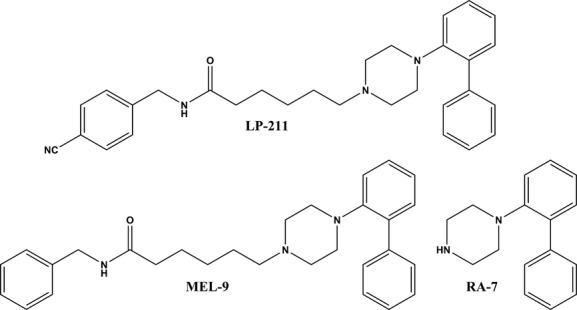
Structures of the arylpiperazine compounds employed in this study.

**Figure 2:**
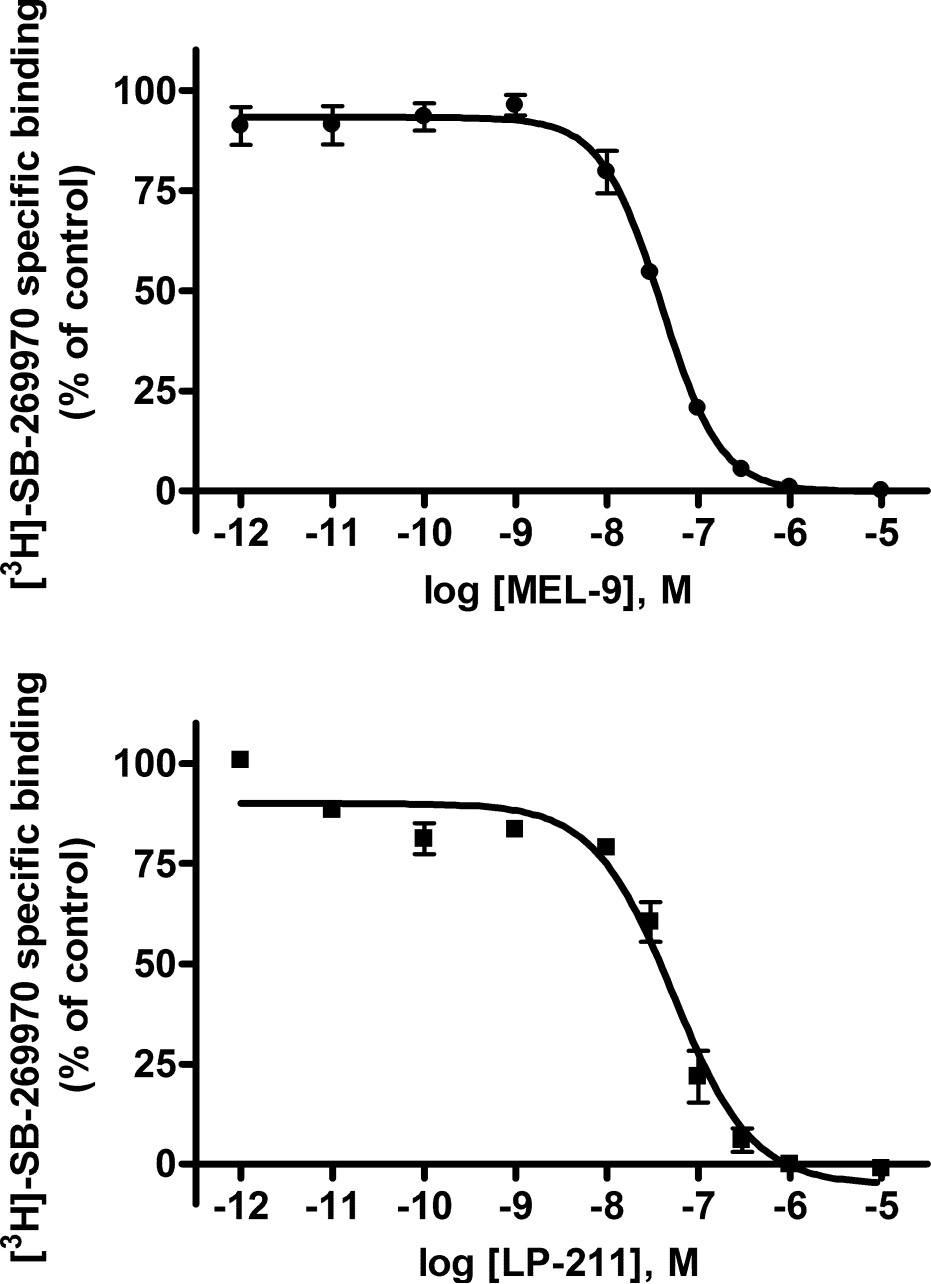
Affinity of compounds for human 5-HT_7_ receptors expressed in HEK293 cells from competitive binding experiments in membrane homogenates. Competition by MEL-9 and LP-211 for binding of [^3^H]-SB-269970 (2 nmol/L) to membrane homogenates. Total radioactivity bound was less than 20% of total radioactivity added in all the experiments. Data are the mean values ± SEM from three independent experiments performed in duplicate. The data were fitted to a sigmoidal dose–response curve (*n*_H_ = 1) or to a sigmoidal dose–response curve (variable slope), according to the best fit model (*P* < 0.05, extra sum-of-squares *F-*test).

Compound LP-211 and its analog MEL-9 (Fig. 1) completely displaced the binding of [^3^H]-SB-269970 to membrane homogenates of HEK293 cells stably expressing the human 5-HT_7(a)_ receptor (HEK-hu5-HT_7_) (Fig. 2), which is compatible with competitive binding. The compounds exhibited affinities in the nanomolar range for human 5-HT_7_ receptors that are consistent with previous findings (Leopoldo et al. 2008; Hedlund et al. 2010), and the Hill slopes (*n*_H_) were close to unity. The affinity constant (*K*_i_, p*K*_i_) and *n*_H_ values for the compounds and methiothepin, included in the experiments as a reference compound, are shown in Table 1.

**Table 1:** Affinity of MEL-9, LP-211 and methiothepin for human 5-HT_7_ receptors expressed in HEK293 cells from [^3^H]-SB-269970 competitive binding experiments

	5-HT_7_ affinity (membrane homogenate radioligand binding)			5-HT_7_ affinity (whole-cell radioligand binding)	
		
Compound	*K*_i_ (nmol/L)	p*K*_i_	*n*_H_	*K*_i_ (nmol/L)	p*K*_i_
MEL-9	16.60 ± 0.99	7.78	1.34 ± 0.14	13.77 ± 0.24	7.86
LP-211	22.52 ± 3.60	7.65	1	19.01 ± 0.14	7.72
Methiothepin	2.31 ± 0.02	8.64	1	2.51 ± 0.73	8.62

The data are the mean values ± SEM from three independent experiments performed in duplicate. MEL-9, *N*-benzyl-4-(2-diphenyl)-1-piperazinehexanamide; LP-211, *N*-(4-cyanophenylmethyl)-4-(2-diphenyl)-1-piperazinehexanamide; HEK293, human embryonic kidney cells; 5-HT_7_, 5-hydroxytryptamine (serotonin) type 7 receptor; [^3^H]-SB-269970, (2*R*)-1-[(3-hydroxyphenyl)sulfonyl]-2-[2-(4-methyl-1-piperidinyl)ethyl]pyrrolidine.

**Figure 3:**
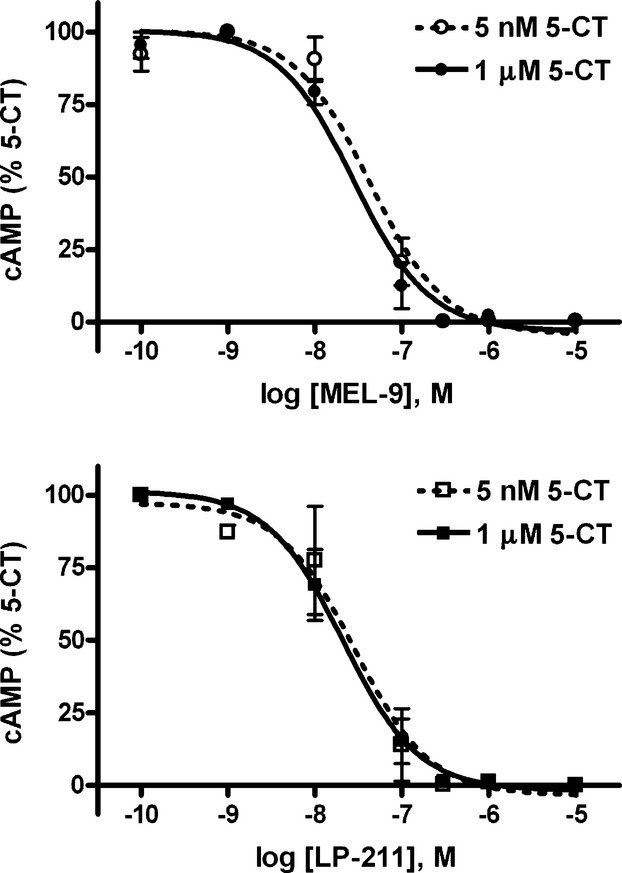
Concentration–response curves for MEL-9 and LP-211 on 5 nmol/L or 1 μmol/L 5-CT-stimulated cAMP production in HEK-hu5-HT_7_ cells. Cells were preincubated in the absence (control) or presence of the compounds at different concentrations (100 pmol/L–10 μmol/L) for 15 min prior to the addition of 5 nmol/L or 1 μmol/L 5-CT and incubation for 15 min. Basal values were subtracted from 5-CT-stimulated values and expressed as percent of the control 5-CT response. 5 nmol/L 5-CT elicited a cAMP response over basal equal to 44.3 ± 1.6% of the response elicited by 1 μmol/L 5-CT. Data are the mean values ± SEM from three independent experiments performed in triplicate.

For characterization of the efficacy profile of the compounds, we evaluated their effects on the human 5-HT_7_-mediated cAMP signaling pathway in HEK-hu5-HT_7_ cells. The compounds did not elicit any detectable increase in cAMP levels in the cells over the range of concentrations assayed (from 100 pmol/L to 10 μmol/L), while the 5-HT_1/7_ receptor-selective agonist 5-CT (100 nmol/L), included in the experiments as a reference agonist, increased cAMP levels by up to more than 50 times above basal levels under the same conditions (data not shown). Moreover, the compounds completely antagonized the cAMP signaling stimulated by 1 μmol/L 5-CT, in a concentration-dependent manner, with IC_50_ values close to their affinities for 5-HT_7_ receptors (IC_50_ = 27.84 ± 6.72 nmol/L and 20.91 ± 5.72 nmol/L, for MEL-9 and LP-211, respectively, *n* = 3). The compounds inhibited with the same potency the cAMP response stimulated by 5 nmol/L and 1 μmol/L 5-CT (Fig. 3), two concentrations of the agonist that differ by ∼200 times (ranging from 0.3 times lower to 69 times higher than the EC_50_ value), therefore showing a behavior not consistent with classical competitive antagonism (Kenakin 2009).

**Figure 4:**
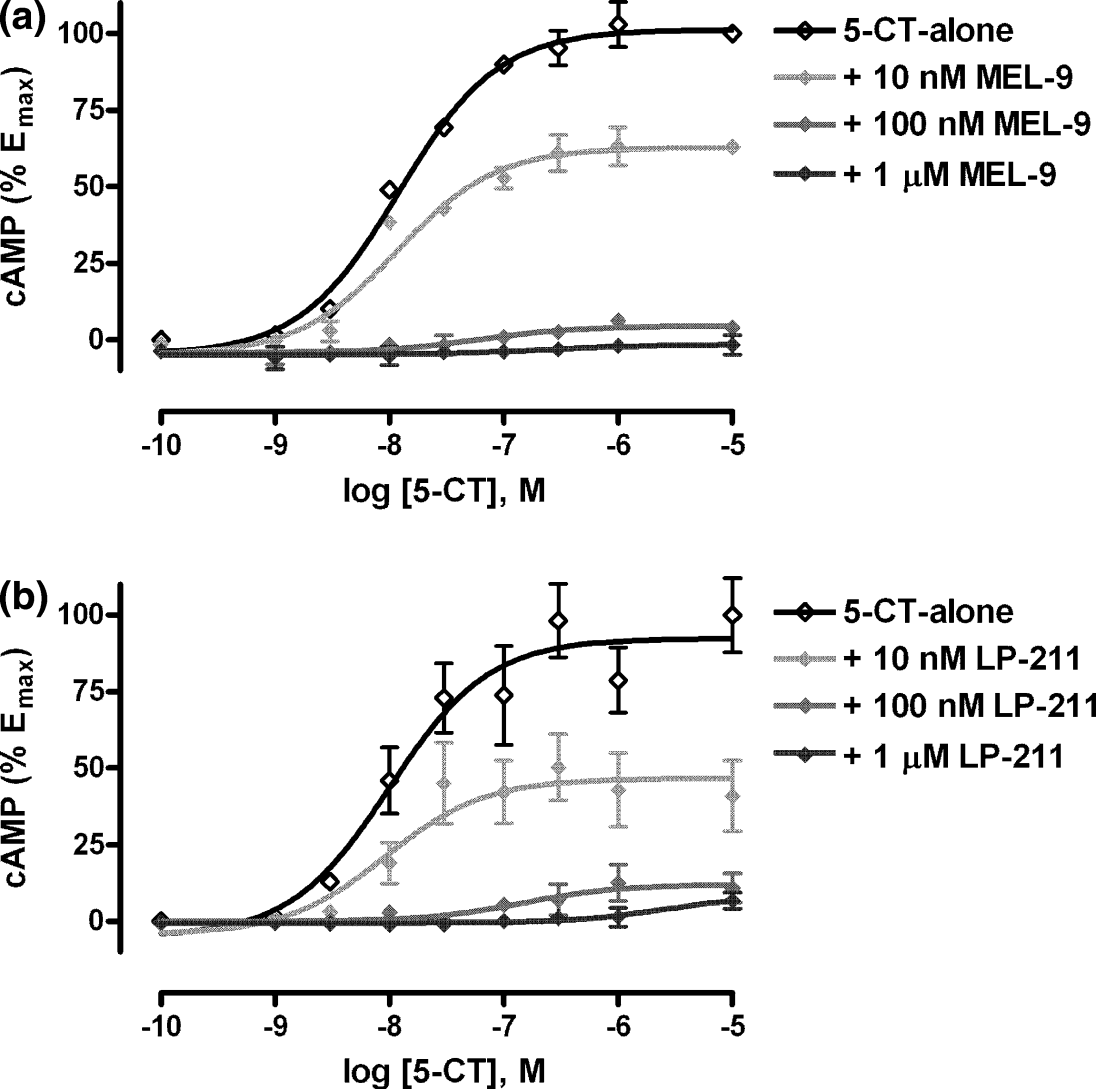
Schild analysis for the antagonism of 5-CT-stimulated cAMP signaling by MEL-9 and LP-211 in HEK-hu5-HT_7_ cells. Concentration–response curves for cAMP production stimulated by 5-CT in the absence (control) or presence of 10 nmol/L, 100 nmol/L or 1 μmol/L of MEL-9 (A) or LP-211 (B). Cells were preincubated with the compounds for 15 min prior to the addition of 5-CT and incubation for 15 min. Basal values were subtracted from 5-CT-stimulated values and expressed as percent of the control maximal 5-CT response. Data are the mean values ± SEM from three independent experiments performed in duplicate.

Concentration–response curves for cAMP production by 5-CT (10 nmol/L–10 μmol/L) in the absence or presence of different concentrations (10 nmol/L, 100 nmol/L or 1 μmol/L) of the antagonists (Schild analysis) revealed an insurmountable antagonistic behavior for both compounds, which produced a rightward shift of the 5-CT concentration–response curves, while reducing the maximal 5-CT response in a concentration-dependent manner until it was completely suppressed (Fig. 4). The EC_50_ values for 5-CT and the percent decrease in the maximal 5-CT response in the absence or presence of different concentrations of the compounds are shown in Table 2.

**Table 2:** Schild analysis for the antagonism of 5-CT-stimulated cAMP signaling by MEL-9 and LP-211 in HEK-hu5-HT_7_ cells

	MEL-9		LP-211	
[Compound]	5-CT EC_50_ (nmol/L)	5-CT *E*_max_ (% reduction)	5-CT EC_50_ (nmol/L)	5-CT *E*_max_ (% reduction)
Control (no compound)	13.34 ± 3.00	0	13.34 ± 3.00	0
10 nmol/L	13.67 ± 1.70	37.10 ± 0.47*	12.24 ± 5.75	58.97 ± 16.42**
100 nmol/L	48.21 ± 6.40	96.02 ± 2.15**	134.7 ± 17.2	89.17 ± 7.31**
1 μmol/L	n.d.	101.6 ± 3.14**	n.d.	93.17 ± 3.87**

The table shows the EC_50_ of 5-CT and the percent reduction in *E*_max_ for each concentration of the compounds studied. Data are the mean values ± SEM from three independent experiments performed in duplicate. (*P < 0.05, **P < 0.01, relative to control; One-way ANOVA and Dunnett's Multiple Comparison Test). n.d., not determined; MEL-9, *N*-benzyl-4-(2-diphenyl)-1-piperazinehexanamide; LP-211, *N*-(4-cyanophenylmethyl)-4-(2-diphenyl)-1-piperazinehexanamide; 5-CT, 5-carboxamidotryptamine; *E*_max_, maximal response.

**Figure 5:**
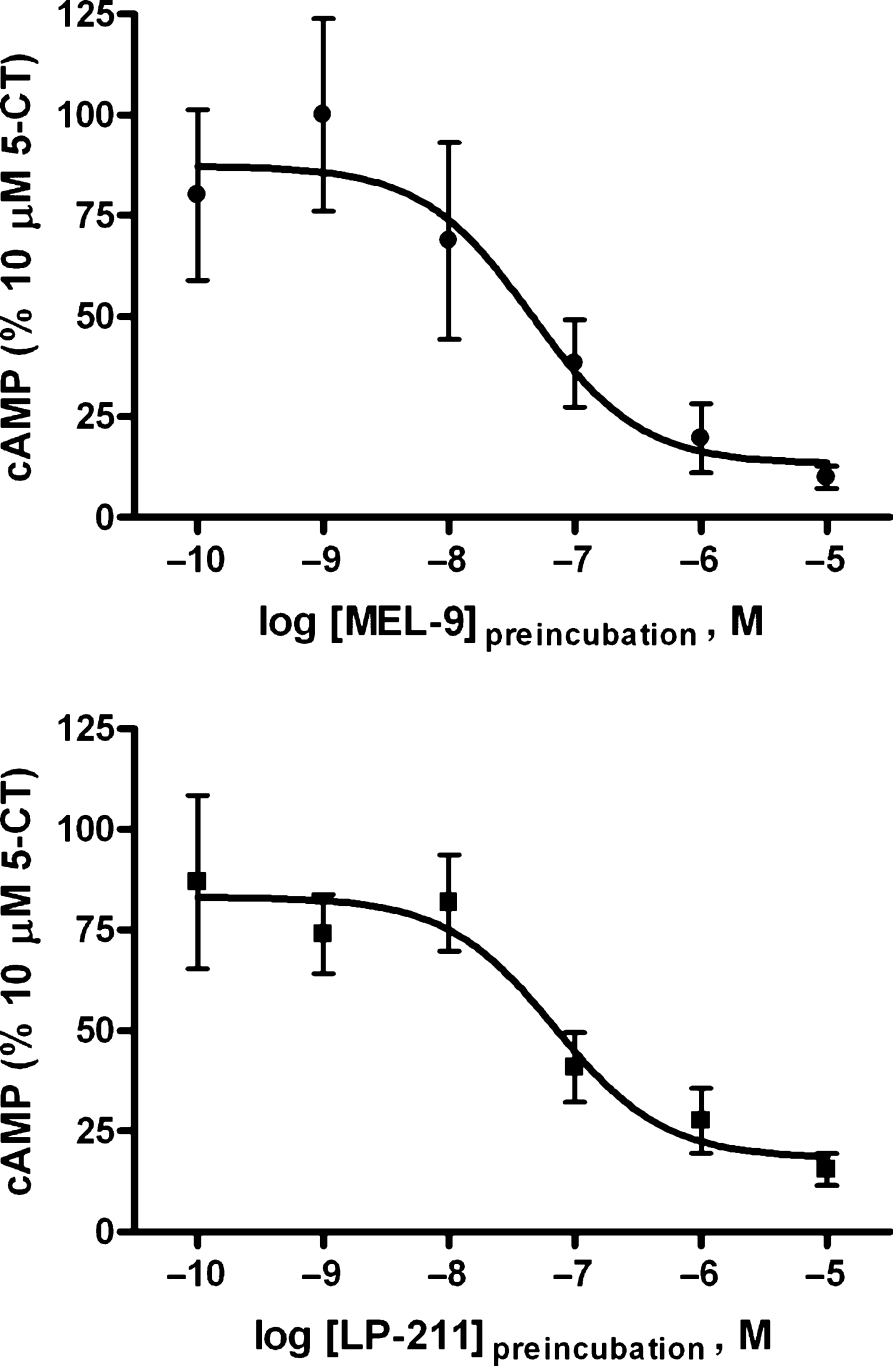
Concentration–response curves for the inhibition of 10 μmol/L 5-CT-stimulated cAMP production by MEL-9 and LP-211 in HEK-hu5-HT_7_ cells in preincubation/washout experiments. Cells were preincubated in the absence (control) or presence of the compounds at increasing concentrations (ranging from 100 pmol/L to 10 μmol/L) for 30 min. After a compound washout procedure (see Materials and Methods), the cells were stimulated with 10 μmol/L 5-CT for 30 min. Basal values were subtracted from 5-CT-stimulated values and expressed as percent of the control 5-CT response. Data are the mean values ± SEM from three independent experiments performed in triplicate.

To analyze the mechanism of the observed insurmountable antagonism, we investigated the reversible or irreversible nature of the action of the compounds at the 5-HT_7_-mediated cAMP pathway by using preincubation/washout experiments. HEK-hu5-HT_7_ cells were treated with no drug (control) or with increasing concentrations of MEL-9 or LP-211 for 30 min before removing the compounds by extensive washout and assessment of the cAMP production stimulated by 5-CT at a concentration of 10 μmol/L (i.e., 690 times higher than the EC_50_). Both compounds induced complete loss of 5-CT-stimulated cAMP signaling (Fig. 5), and the potency for this irreversible inhibition (IC_50, irrev_) was in the nanomolar range (Table 3). This supports an irreversible or pseudo-irreversible mechanism of antagonism by both compounds in the cAMP pathway.

**Table 3:** Potency of MEL-9 and LP-211 as irreversible inhibitors of 5-CT-stimulated cAMP signaling and of whole-cell [^3^H]-SB-269970 radioligand binding in HEK-hu5-HT_7_ cells in preincubation/washout experiments

Potency as irreversible inhibitors of human 5-HT_7_ receptors in preincubation/washout experiments		
	5-CT-stimulated cAMP production	Whole-cell [^3^H]-SB-269970 radioligand binding
Compound	IC_50, irrev_ (nmol/L)	IC_50, irrev_ (nmol/L)
MEL-9	41.84 ± 18.03	22.42 ± 7.63
LP-211	101.65 ± 38.00	76.26 ± 25.39

IC_50, irrev_ denotes the potency of the compounds as irreversible inhibitors of 5-HT_7_-mediated cAMP signaling or whole-cell 5-HT_7_ radioligand binding in assays carried out after preincubation of the cells with the compounds and subsequent compound washout. Data are the mean values ± SEM from three independent experiments performed in triplicate (cAMP assays) or of three independent experiments performed in duplicate (whole-cell radioligand binding). MEL-9, *N*-benzyl-4-(2-diphenyl)-1-piperazinehexanamide; LP-211, *N*-(4-cyanophenylmethyl)-4-(2-diphenyl)-1-piperazinehexanamide; 5-CT, 5-carboxamidotryptamine; [^3^H]-SB-269970, (2*R*)-1-[(3-hydroxyphenyl)sulfonyl]-2-[2-(4-methyl-1-piperidinyl)ethyl]pyrrolidine; 5-HT_7_, 5-hydroxytryptamine (serotonin) type 7 receptor.

**Figure 6:**
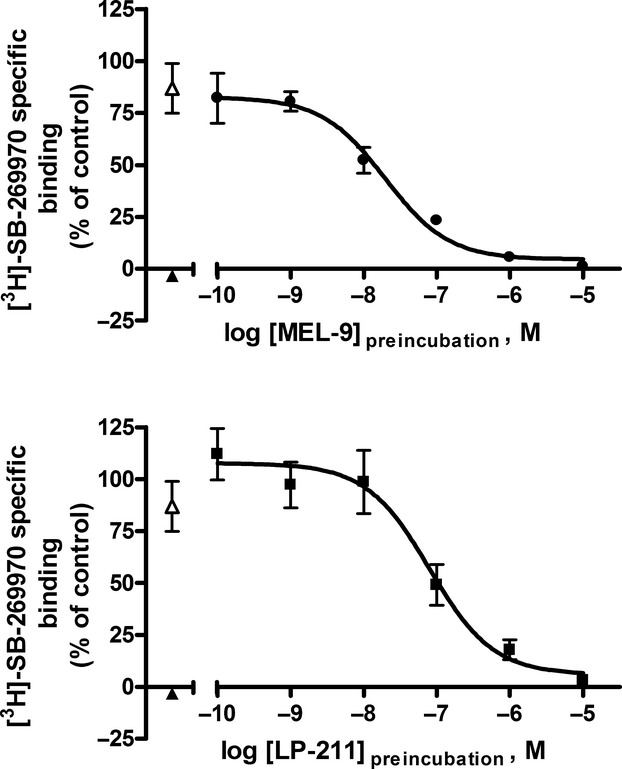
Concentration–response curves for the inhibition of 5-HT_7_ binding sites by MEL-9 and LP-211 in preincubation/washout experiments in radioligand binding studies in whole HEK-hu5-HT_7_ cells. Cells were preincubated in the absence (control) or presence of either MEL-9 or LP-211 at increasing concentrations (ranging from 100 pmol/L to 10 μmol/L), 10 μmol/L clozapine (Δ) as reference competitive antagonist, or 1 μmol/L methiothepin (▲) as reference irreversible antagonist, for 30 min. After a compound washout procedure (see Materials and Methods), the cells were subjected to whole-cell radioligand binding assays to determine specific [^3^H]-SB-269970 binding. [^3^H]-SB-269970 specific binding is expressed in each case as percent of [^3^H]-SB-269970 specific binding in cells preincubated in the absence of compound (control). The total radioactivity bound was less than 20% of total radioactivity added in all the experiments. Data are the mean values ± SEM from three to five independent experiments performed in duplicate. Concentration–response data were fitted to a sigmoidal dose–response curve (*n*_H_ = 1).

In light of our previous results, we investigated a possible irreversible or pseudo-irreversible interaction between the compounds and the human 5-HT_7_ receptor. MEL-9 and LP-211 fully displaced the binding of [^3^H]-SB-269970 to 5-HT_7_ receptors in competition binding experiments performed with whole-HEK-hu5-HT_7_ cells, exhibiting affinities in the range of those obtained in cell membrane homogenate binding assays (Table 1). The same was observed for methiothepin, included in the experiments as a reference compound (Table 1). Preincubation of the cells with increasing concentrations (100 pmol/L–10 μmol/L) of MEL-9 or LP-211, followed by washout of the compounds and subsequent [^3^H]-SB-269970 radioligand binding to the intact cells resulted in a complete concentration-dependent reduction in [^3^H]-SB-269970 specific binding. Preincubation of the cells with 1 μmol/L methiothepin also led to the complete loss of [^3^H]-SB-269970 specific binding ([^3^H]-SB-269970 specific binding, expressed as percent of the [^3^H]-SB-269970 specific binding in cells preincubated in the absence of compound (control) = −3.27 ± 1.06%, *n* = 5). [^3^H]-SB-269970 specific binding in cells preincubated with 10 μmol/L clozapine was, however, 77.74 ± 11.57% (*n* = 5) of that in cells preincubated in the absence of compound (control) (Fig. 6). These results indicate irreversible inhibition of binding at 5-HT_7_ binding sites by the compounds, parallel to the observed inhibition of the cAMP signaling. The potency of the compounds as irreversible inhibitors of 5-HT_7_ radioligand binding observed in these experiments, expressed as the concentration of compound in the preincubation step that reduces the [^3^H]-SB-269970 specific binding by 50% in intact cells following removal of the compounds (IC_50, irrev_), is shown in Table 3.

**Figure 7:**
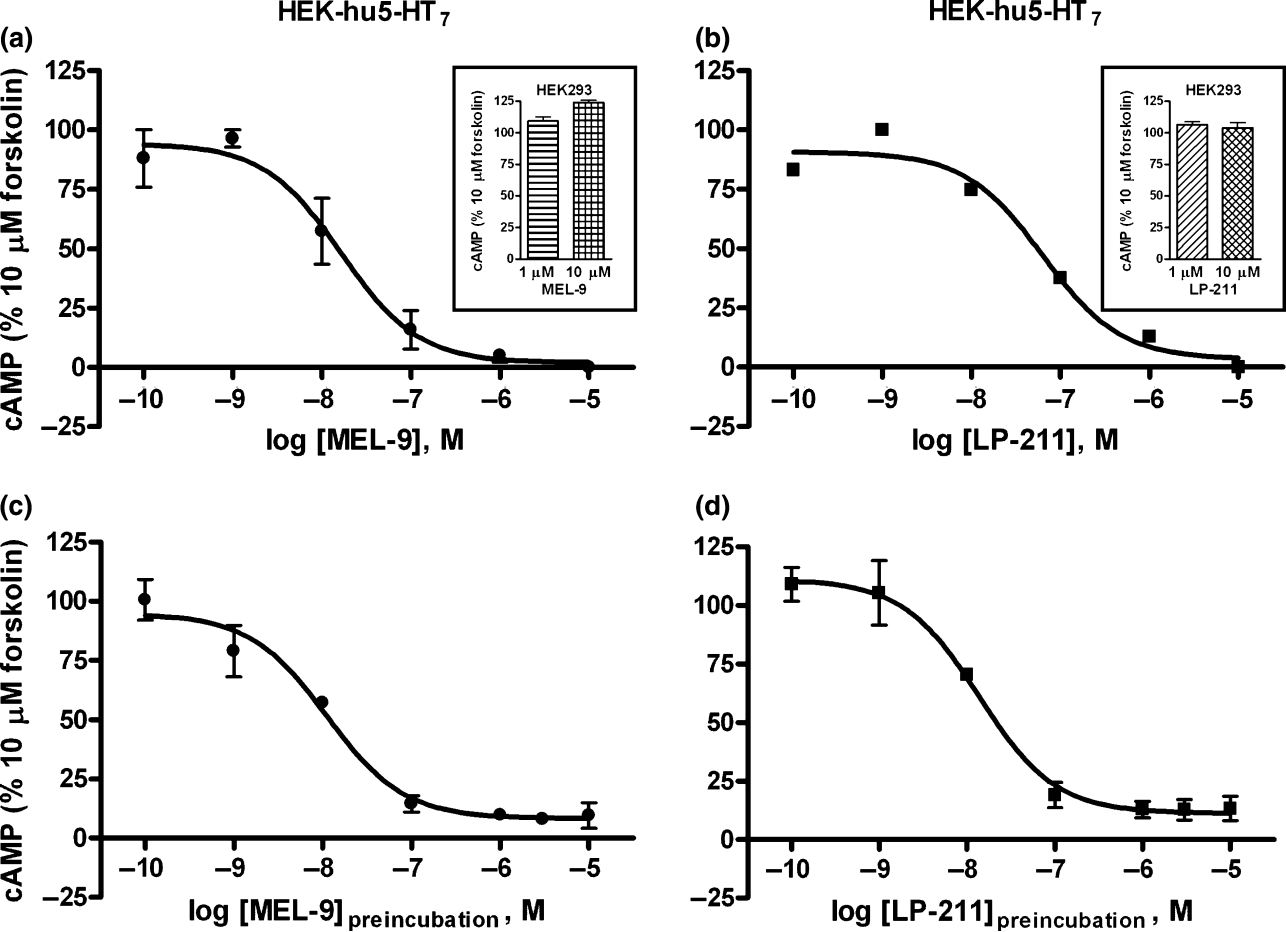
Effect of compounds MEL-9 and LP-211 on cAMP signaling stimulated by forskolin in HEK-hu5-HT_7_ cells. (A, B) Concentration–response curves for MEL-9 (A) and LP-211 (B) on 10 μmol/L forskolin-stimulated cAMP production. Cells were preincubated in the absence (control) or presence of the compounds at different concentrations (100 pmol/L – 10 μmol/L) for 15 min prior to the addition of 10 μmol/L forskolin and incubation for 15 min. Basal values were subtracted from forskolin-stimulated values and expressed as percent of the control forskolin response. Data are the mean values ± SEM from three independent experiments performed in triplicate. (A, B Inset) Lack of effect of 1 μmol/L or 10 μmol/L MEL-9 (A, Inset) and 1 μmol/L or 10 μmol/L LP-211 (B, Inset) on forskolin-stimulated cAMP production in parental HEK293 cells. (C, D) Concentration–response curves for the inhibition of 10 μmol/L forskolin-stimulated cAMP production by MEL-9 (C) and LP-211 (D) in HEK-hu5-HT_7_ cells in preincubation/washout experiments. Cells were preincubated in the absence (control) or presence of the compounds at increasing concentrations (ranging from 100 pmol/L to 10 μmol/L) for 30 min. After a compound washout procedure (see Materials and Methods), the cells were stimulated with 10 μmol/L forskolin for 15 min. Basal values were subtracted from forskolin-stimulated values and expressed as percent of the control forskolin response. Data are the mean values ± SEM from three independent experiments performed in triplicate.

Inhibition of forskolin-stimulated adenylate cyclase activity by 5-HT_7_ antagonists was previously reported in transfected cell lines (Klein and Teitler 2011). We investigated a possible effect of MEL-9 and LP-211 on forskolin-stimulated adenylate cyclase activity. Both MEL-9 and LP-211 fully inhibited the 10 μmol/L forskolin-stimulated cAMP production in HEK-hu5-HT_7_ cells, in a concentration-dependent manner, when forskolin was assayed in the continuous presence of the compounds (Fig. 7A and B). The compounds showed no effect on forskolin-stimulated cAMP production in the parental cell line (Fig. 7A and B, inset). The potencies of the compounds as inhibitors of forskolin-stimulated adenylate cyclase activity were in the range of their affinities for 5-HT_7_ receptors (IC_50_ = 19.33 ± 8.18 nmol/L and 60.71 ± 0.48 nmol/L, for MEL-9 and LP-211, respectively).

Moreover, washout of the compounds for 30 min prior to stimulation of the cells with forskolin did not lead to recovery of the forskolin-stimulation of adenylate cyclase activity, but to long-lasting concentration-dependent inhibition of forskolin effects (Fig. 7C and D). The potency of the compounds as irreversible inhibitors of forskolin-stimulated adenylate cyclase activity observed in these experiments, expressed as the concentration of compound in the preincubation step that reduces the forskolin-stimulation of adenylate cyclase to 50% following removal of the compounds, was IC_50, irrev_ = 11.17 ± 3.25 nmol/L and 13.95 ± 3.93 nmol/L, for MEL-9 and LP-211, respectively (*n* = 3).

**Figure 8:**
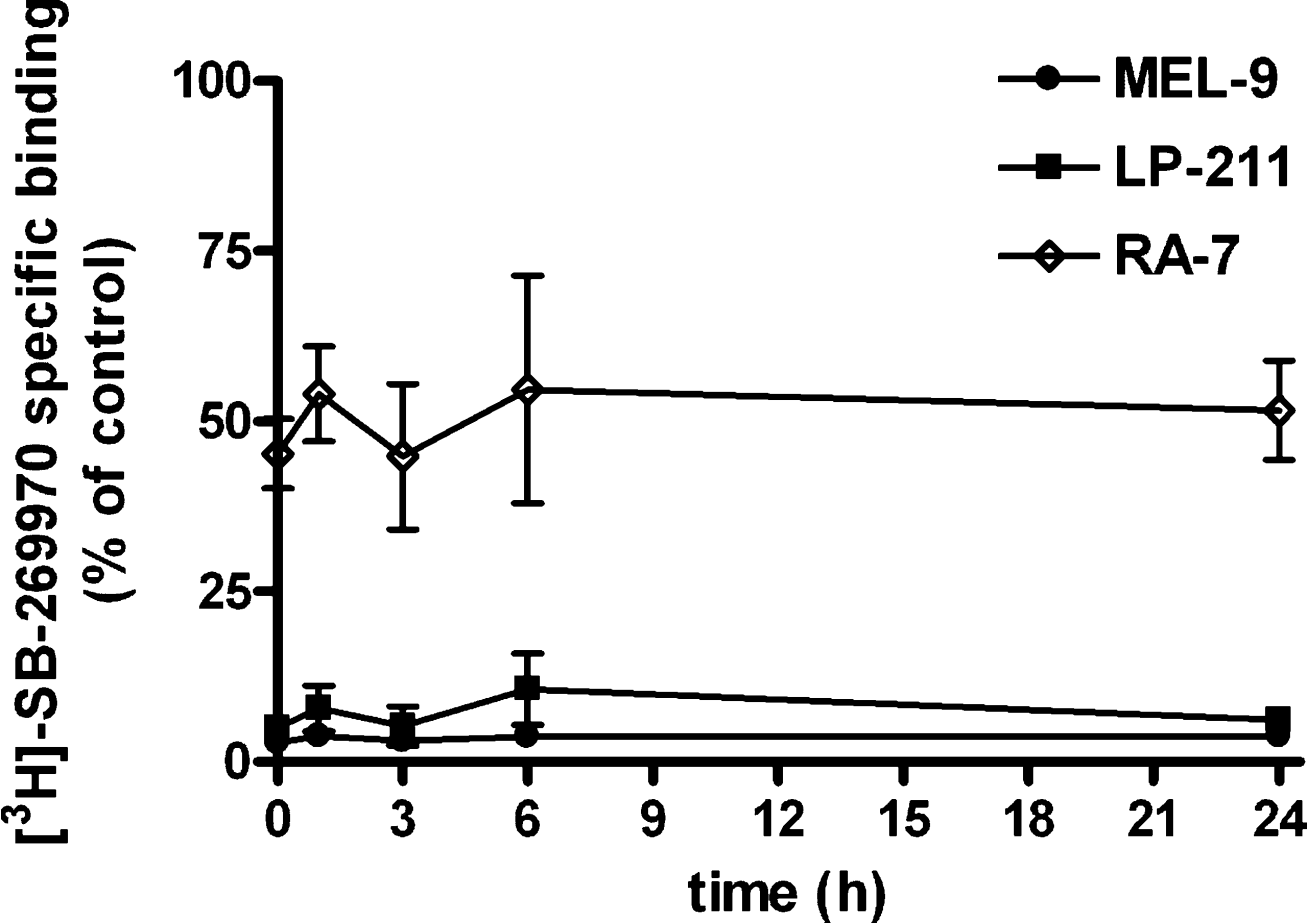
Long-lasting inhibition of [^3^H]-SB-269970-binding sites by MEL-9 and LP-211 in HEK-hu5-HT_7_ cells in preincubation/washout experiments. Cells were preincubated in the absence (control) or presence of 10 μmol/L MEL-9, 10 μmol/L LP-211, or 10 μmol/L RA-7 for 30 min. After a compound washout procedure (see Materials and Methods), cells were further incubated at 37°C for the indicated times before being subjected to whole-cell radioligand binding assays for determination of specific [^3^H]-SB-269970 binding. Total radioactivity bound was less than 20% of total radioactivity added in all the experiments. Specific binding is expressed as percent of the specific binding of control at each time point. Data are the mean values ± SEM from three independent experiments performed in duplicate.

Finally, we determined the time course of recovery of specific binding of [^3^H]-SB-269970 to 5-HT_7_ receptors after removal of the compounds. Whole-cell radioligand binding assays performed in HEK-hu5-HT_7_ cells at different times after preincubation of the cells with the compounds at a maximal inhibitory concentration (10 μmol/L) and subsequent removal of the compounds revealed long-lasting inhibition of [^3^H]-SB-269970 binding to 5-HT_7_ receptors by both compounds, which did not recover after 24 h (Fig. 8).

In a recent work from our group, we investigated the agonist or antagonist action of a set of unsubstituted 1-(biphenyl)piperazine derivatives at 5-HT_7_ receptors (Lacivita et al. 2012). The study revealed that compound RA-7 (Fig. 1) was a high-affinity receptor 5-HT_7_ antagonist in HeLa cells expressing the receptor (Lacivita et al. 2012). This compound attracted our attention while performing this study because disposition studies in mice revealed that LP-211 undergoes N-dealkylation to RA-7 (Hedlund et al. 2010). Therefore, we aimed to investigate the possible contribution of the RA-7 moiety to the long-lasting irreversible inhibition of 5-HT_7_ binding sites by MEL-9 and LP-211. RA-7 (10 μmol/L) fully displaced the binding of [^3^H]-SB-269970 to 5-HT_7_ receptors in competitive binding experiments performed in whole-HEK-hu5-HT_7_ cells ([^3^H]-SB-269970 specific binding (percent of control) = 0.64 ± 0.44%, *n* = 3), consistent with the previously reported affinity of RA-7 at human 5-HT_7_ receptors (Hedlund et al. 2010). Preincubation of HEK-hu5-HT_7_ cells for 30 min at 37°C with 10 μmol/L RA-7 and subsequent compound washout led to inhibition of the specific binding of [^3^H]-SB-269970 to 5-HT_7_ receptors of 54.76 ± 5.15% at time *t* = 0 (Fig. 8); this was significantly lower than the reduction of 97.20 ± 0.23% and 95.03 ± 0.47% achieved by MEL-9 and LP-211 at equimolar concentrations, respectively (*P* < 0.001, One-way analysis of variance (ANOVA) and Bonferroni's Multiple Comparison Test). However, and similar to what was observed for MEL-9 and LP-211, the partial inhibition of [^3^H]-SB-269970 binding to 5-HT_7_ receptors elicited by RA-7 was also long-lasting (it lasted for at least 24 h) (Fig. 8). The results indicate the contribution of the *N*-phenylmethyl-hexenamide moiety to the efficacy of the compounds as irreversible inhibitors of 5-HT_7_-binding sites and the ability of the biphenyl-piperazine moiety to confer the compounds with the capacity to inhibit the 5-HT_7_ binding in the long term.

## Discussion

The *K*_i_ value of LP-211 for human 5-HT_7_ receptors obtained in this study confirms our previous reports (Hedlund et al. 2010). In the case of MEL-9, we observed full displacement of [^3^H]-SB-269970 binding in this study as well as of [^3^H]-LSD (lysergic acid diethylamide) binding (*K*_i_ = 37.92 ± 11.30 nmol/L, *n* = 3, *n*_H_ = 1.11 ± 0.19) (data not shown) in membrane homogenates of HEK-hu5-HT_7_ cells. The difference between the present results and those previously reported for MEL-9 (Leopoldo et al. 2008) may be due to the different cell lines or binding protocols used in each case. The reported affinities of MEL-9 for 5-HT_1A_ and D_2_ receptors were 9.7-fold and 30-fold lower, respectively, than its affinity for 5-HT_7_ receptors obtained in our study (Leopoldo et al. 2008). On the other hand, SB-269970 is considered to date the standard selective 5-HT_7_ antagonist and a prototype 5-HT_7_ radioligand (Hagan et al. 2000; Thomas et al. 2000). SB-269970 displays a 50-fold selectivity versus 5-ht_5_ receptors, a receptor subtype reported not to be endogenously expressed at significant levels in HEK293 cells (Atwood et al. 2011), and over 100-fold selectivity versus other 5-HT receptors, dopaminergic D_2_ or D_3_ receptors, and adrenergic alpha_1b_ receptors (Lovell et al. 2000). Considering the affinity profile of the ligands used in this study, the effects of MEL-9 and LP-211 on the [^3^H]-SB-269970 binding appear to originate from their specific interaction with 5-HT_7_ receptors.

MEL-9 and LP-211 behaved as irreversible or pseudo-irreversible inhibitors of 5-HT_7_-binding sites in preincubation/washout experiments. Direct assessment of whether the compounds remain irreversibly bound to the receptor and whether the long-lasting inhibition of binding sites persists after compound dissociation requires a labeled form of the compounds not available to us at this stage. Methiothepin, a so-called “inactivating antagonist” of 5-HT_7_ receptors (Smith et al. 2006), at the concentration of 1 μmol/L (∼270 times its *K*_i_ at human 5-HT_7_ receptors) also completely inhibit 5-HT_7_-binding sites under our experimental conditions, while the [^3^H]-SB-269970 specific binding in cells preincubated with the competitive 5-HT_7_ antagonist clozapine at the concentration of 10 μmol/L (∼500 times its *K*_i_ at human 5-HT_7_ receptors) was preserved to 78% of control. This fact provides compelling evidence that any residual compound remaining after insufficient washing does not mediate the inhibitory effects of MEL-9 or LP-211. The same reasoning may apply to compound RA-7, which caused a decrease of ∼50% in the [^3^H]-SB-269970 specific binding on preincubation at a concentration equimolar to that of MEL-9 and LP-211. Furthermore, considering that the affinity of RA-7 for 5-HT_7_ receptors has been reported to be higher than that of MEL-9 and LP-211 (Hedlund et al. 2010), receptor occupancy by itself is not sufficient to explain the efficacy of the compounds inhibiting [^3^H]-SB-269970 binding.

Incubation of intact cells with agonists often results in internalization of G protein-coupled receptors (GPCR) away from the plasma membrane (Moore et al. 2007). GPCR internalization has been reported to be dissociated from signaling for certain GPCR ligands (Gray and Roth 2001; Sneddon et al. 2004). Hence, the loss of [^3^H]-SB-269970 binding in intact cells following preincubation with MEL-9 and LP-211 may be attributed to receptor internalization, even in the absence of cAMP signaling elicited by the compounds. Hydrophobic radioligands such as [^125^I]-iodopindolol (*x*log *P* = 2.99) and [^3^H]-mesulergine (*x*log *P* = 1.80) successfully labeled beta-adrenergic and 5-HT_7_ receptors, respectively, both at the cell surface and within endocytotic vesicles in intact cells (Reynolds and Molinoff 1986; Andressen et al. 2006). On the basis of the lipophilicity of [^3^H]-SB-269970 (*x*log *P* = 2.59), we would expect this radioligand to label both cell surface and internalized 5-HT_7_ receptors in equilibrium binding assays with intact cells. This precludes receptor internalization as a possible explanation for the loss of radioligand binding following preincubation of intact cells with the compounds under study. Receptor downregulation, that is, a reduction in the total number of receptors, generally requires repeated or prolonged (typically for several hours) activation of the receptors (Tsao and von Zastrow 2000), and it does not usually lead to the complete abolition of receptor binding. Downregulation of the 5-HT_7_ receptor was not observed in HEK293 or Caco-2 cells after treatment with agonists or antagonists for periods longer than 30 min, even under conditions that led to receptor desensitization (Krobert et al. 2006; Iceta et al. 2009). Therefore, it appears unlikely that receptor downregulation accounts for the complete abolition of [^3^H]-SB-269970 binding observed in this study.

The observed inhibition of the 5-CT-stimulated cAMP signaling by the compounds was unexpected, particularly for LP-211, which has been extensively studied to date and to which 5-HT_7_ agonist activity has been attributed in ex vivo and in vivo studies. Hence, LP-211 elicited relaxation of substance P-induced contraction in isolated guinea pig ileum (Leopoldo et al. 2008), induced hypothermia in wild type but not in 5-HT_7_ knock-out mice (Hedlund et al. 2010), and it reverted long-term synaptic depression induced by metabotropic glutamate receptors in mouse hippocampal slices (Costa et al. 2012). These effects are consistent with activation of 5-HT_7_ receptors by LP-211. Nevertheless, LP-211 exerted antianxiety-like effects in mice, similar to those described for the 5-HT_7_ antagonist SB-269970 (Adriani et al. 2012). Full interpretation of these studies would require a better understanding of how 5-HT_7_ receptor modulation in vivo contributes to the observed effects through direct versus indirect mechanisms. In particular, the consequences of 5-HT_7_ receptor activation on 5-HT release and serotonergic neural activity may depend on endogenous 5-HT levels (Matthys et al. 2011).

One possible explanation for the irreversible inhibition of the 5-HT_7_-mediated cAMP pathway by both compounds is that preincubation with the compounds led to desensitization of 5-HT_7_ receptors. This should involve a cAMP-independent mechanism, taking into account the lack of cAMP stimulation elicited by the compounds. Recent studies reported desensitization of 5-HT_7_-mediated adenylate cyclase activity in membranes from HEK293 cells through a protein kinase A-independent mechanism, after preincubation of the intact cells for 1 h with SB-269970 (Krobert et al. 2006). SB-269970 also elicited functional desensitization of 5-HT_7_ receptors in acute administration in vivo (Tokarski et al. 2012). However, the mechanism responsible for the reduction in 5-HT_7_ agonist-mediated signaling by SB-269970 appears to differ from that operating in the case of MEL-9 and LP-211. First, the reduction did not achieve 100% of the agonist signal in either of the two studies mentioned, and second, contrary to what we observed, desensitization did not occur in parallel to the loss of 5-HT_7_-binding sites. 5-HT_7_ receptors have shown characteristics consistent with a model in which an inactive conformational state of the receptor is tightly associated with G_s_ protein, independent of agonist binding (Bruheim et al. 2003; Andressen et al. 2006). Therefore, a possible explanation for the inhibitory effects of the compounds on 5-HT_7_-mediated cAMP signaling is that the compounds irreversibly stabilize this receptor conformation.

The inhibition of forskolin-stimulated adenylate cyclase activity by MEL-9 and LP-211 specifically in HEK-hu5-HT_7_ cells and not in the parental cell line reinforces the idea that the effects are due to specific interactions of the compounds with 5-HT_7_ receptors, ruling out nonspecific effects or a direct interaction with the adenylate cyclase enzyme as mechanisms responsible for the effect. This inhibition was also observed in preincubation/wash-out experiments, as expected for the so-called 5-HT_7_ “inactivating antagonists” (Toohey et al. 2009). 5-HT_7_ pre-associated signaling complexes may include receptor, G protein and adenylate cyclase (Bruheim et al. 2003). By interacting with the receptor, the “inactivating” compounds may alter the G_s_/adenylate cyclase interaction in a way that persistently hinders the action of forskolin on adenylate cyclase. Another possible explanation for this effect of the compounds is that they might act as 5-HT_7_-biased agonists that activate G_i_ signaling leading to inhibition of forskolin-stimulated cAMP accumulation. However, this seems improvable as G_i_-coupling was not reported for this receptor and this mechanism was ruled out both for 5-HT_7_ “inactivating antagonists” and competitive antagonists by using pertussis toxin (Toohey et al. 2009; Klein and Teitler 2011).

In conclusion, the results allow us to define MEL-9 and LP-211 as irreversible inhibitors of 5-HT_7_ receptors, being the first report of arylpiperazine derivatives as long-lasting inhibitors of 5-HT_7_ receptors. Our data are consistent with previous observations of irreversible “inactivation” of 5-HT_7_ receptors by certain multitarget drugs (Smith et al. 2006; Toohey et al. 2009). Overall, the present results open up new perspectives on the study of 5-HT_7_ receptors. First, to the best of our knowledge, LP-211 and MEL-9 are the first selective 5-HT_7_ receptor long-lasting inhibitors to be available. Second, the different profile exhibited by LP-211 and MEL-9 as compared to RA-7 suggests that the feature of irreversible or pseudo-irreversible inhibition of 5-HT_7_ binding sites is structure-dependent. Third, as LP-211 has displayed a behavior in 5-HT_7_ receptor-expressing HEK-293 cells different to that shown in other systems ex vivo and in vivo (i.e., antagonism vs. agonism), questions raise about interpretation of the data obtained by using LP-211. Addressing the above issues will be extremely useful for elucidating the physiological significance of the 5-HT_7_ receptor.

## Abbreviations

5-CT: 5-carboxamidotryptamine
5-HT: 5-hydroxytryptamine (serotonin)
ANOVA: analysis of variance
*E*_max_: maximal response
Fmr1: fragile X mental retardation 1
Fos: FBJ murine osteosarcoma viral oncogene homolog
GPCR: G protein-coupled receptors
HEK293: human embryonic kidney cells
HTRF: homogeneous time-resolved fluorescence
IBMX: 3-isobutyl-1-methylxanthine
IC_50, irrev_: potency of compounds inhibiting 5-HT_7_-stimulated cAMP signaling or whole-cell 5-HT_7_ radioligand binding in preincubation/washout experiments
LP-211: *N*-(4-cyanophenylmethyl)-4-(2-diphenyl)-1-piperazinehexanamide
LSD: lysergic acid diethylamide
MEL-9: *N*-benzyl-4-(2-diphenyl)-1-piperazinehexanamide
RA-7: 1-(2-biphenyl)piperazine
REM: rapid eye movement
SB-269970: (2*R*)-1-[(3-hydroxyphenyl)sulfonyl]-2-[2-(4-methyl-1-piperidinyl)ethyl]pyrrolidine
SERT: serotonin transporter
*x*log *P*: prediction of log of octanol/water partition coefficient

## References

[b1] Adriani W, Travaglini D, Lacivita E, Saso L, Leopoldo M, Laviola G (2012). Modulatory effects of two novel agonists for serotonin receptor 7 on emotion, motivation and circadian rhythm profiles in mice. Neuropharmacology.

[b2] Andressen KW, Norum JH, Levy FO, Krobert KA (2006). Activation of adenylyl cyclase by endogenous G(s)-coupled receptors in human embryonic kidney 293 cells is attenuated by 5-HT(7) receptor expression. Mol Pharmacol.

[b3] Atwood BK, Lopez J, Wager-Miller J, Mackie K, Straiker A (2011). Expression of G protein-coupled receptors and related proteins in HEK293, AtT20, BV2, and N18 cell lines as revealed by microarray analysis. BMC Genomics.

[b4] Bonaventure P, Dugovic C, Kramer M, De Boer P, Singh J, Wilson S (2012). Translational evaluation of JNJ-18038683, a 5-hydroxytryptamine type 7 receptor antagonist, on rapid eye movement sleep and in major depressive disorder. J Pharmacol Exp Ther.

[b5] Brenchat A, Romero L, García M, Pujol M, Burgueño J, Torrens A (2009). 5-HT7 receptor activation inhibits mechanical hypersensitivity secondary to capsaicin sensitization in mice. Pain.

[b6] Bruheim S, Krobert KA, Andressen KW, Levy FO (2003). Unaltered agonist potency upon inducible 5-HT7(a) but not 5-HT4(b) receptor expression indicates agonist-independent association of 5-HT7(a) receptor and Gs. Receptors Channels.

[b7] Cates LN, Roberts AJ, Huitron-Resendiz S, Hedlund PB (2013). Effects of lurasidone in behavioral models of depression. Role of the 5-HT7 receptor subtype. Neuropharmacology.

[b8] Costa L, Spatuzza M, D'Antoni S, Bonaccorso CM, Trovato C, Musumeci SA (2012). Activation of 5-HT7 serotonin receptors reverses metabotropic glutamate receptor-mediated synaptic plasticity in wild-type and Fmr1 knockout mice, a model of Fragile X syndrome. Biol Psychiatry.

[b9] Gray JA, Roth BL (2001). Paradoxical trafficking and regulation of 5-HT(2A) receptors by agonists and antagonists. Brain Res Bull.

[b10] Hagan JJ, Price GW, Jeffrey P, Deeks NJ, Stean T, Piper D (2000). Characterization of SB-269970-A, a selective 5-HT(7) receptor antagonist. Br J Pharmacol.

[b11] Hedlund PB (2009). The 5-HT7 receptor and disorders of the nervous system: an overview. Psychopharmacology.

[b12] Hedlund PB, Sutcliffe JG (2004). Functional, molecular and pharmacological advances in 5-HT7 receptor research. Trends Pharmacol Sci.

[b13] Hedlund PB, Leopoldo M, Caccia S, Sarkisyan G, Fracasso C, Martelli G (2010). LP-211 is a brain penetrant selective agonist for the serotonin 5-HT(7) receptor. Neurosci Lett.

[b14] Henigsberg N, Mahableshwarkar AR, Jacobsen P, Chen Y, Thase ME (2012). A randomized, double-blind, placebo-controlled 8-week trial of the efficacy and tolerability of multiple doses of Lu AA21004 in adults with major depressive disorder. J Clin Psychiatry.

[b15] Hoyer D, Hannon JP, Martin GR (2002). Molecular, pharmacological and functional diversity of 5-HT receptors. Pharmacol Biochem Behav.

[b16] Iceta R, Mesonero JE, Aramayona JJ, Alcalde AI (2009). Expression of 5-HT1A and 5-HT7 receptors in Caco-2 cells and their role in the regulation of serotonin transporter activity. J Physiol Pharmacol.

[b17] Irwin JJ, Sterling T, Mysinger MM, Bolstad ES, Coleman RG (2012). ZINC: a free tool to discover chemistry for biology. J Chem Inf Model.

[b18] Jasper JR, Kosaka A, To ZP, Chang DJ, Eglen RM (1997). Cloning, expression and pharmacology of a truncated splice variant of the human 5-HT7 receptor (h5-HT7b). Br J Pharmacol.

[b19] Kenakin TP (2009). A pharmacology primer. theory, applications, and methods.

[b20] Klein MT, Teitler M (2011). Antagonist interaction with the human 5-HT(7) receptor mediates the rapid and potent inhibition of non-G-protein-stimulated adenylate cyclase activity: a novel GPCR effect. Br J Pharmacol.

[b21] Krobert KA, Andressen KW, Levy FO (2006). Heterologous desensitization is evoked by both agonist and antagonist stimulation of the human 5-HT(7) serotonin receptor. Eur J Pharmacol.

[b22] Lacivita E, Patarnello D, Stroth N, Caroli A, Niso M, Contino M (2012). Investigations on the 1-(2-biphenyl)piperazine motif: identification of new potent and selective ligands for the serotonin(7) (5-HT(7)) receptor with agonist or antagonist action in vitro or ex vivo. J Med Chem.

[b23] Lemoine L, Andries J, Le Bars D, Billard T, Zimmer L (2011). Comparison of 4 radiolabeled antagonists for serotonin 5-HT(7) receptor neuroimaging: toward the first PET radiotracer. J Nucl Med.

[b24] Leopoldo M, Berardi F, Colabufo NA, Contino M, Lacivita E, Niso M (2004a). Structure-affinity relationship study on N-(1,2,3,4-tetrahydronaphthalen-1-yl)-4-aryl-1-piperazinealkylamides, a new class of 5-hydroxytryptamine7 receptor agents. J Med Chem.

[b25] Leopoldo M, Berardi F, Colabufo NA, Contino M, Lacivita E, Perrone R (2004b). Studies on 1-arylpiperazine derivatives with affinity for rat 5-HT7 and 5-HT1A receptors. J Pharm Pharmacol.

[b26] Leopoldo M, Lacivita E, Contino M, Colabufo NA, Berardi F, Perrone R (2007). Structure-activity relationship study on N-(1,2,3,4-tetrahydronaphthalen-1-yl)-4-aryl-1-piperazinehexanamides, a class of 5-HT7 receptor agents. 2. J Med Chem.

[b27] Leopoldo M, Lacivita E, De Giorgio P, Fracasso C, Guzzetti S, Caccia S (2008). Structural modifications of N-(1,2,3,4-tetrahydronaphthalen-1-yl)-4-aryl-1-piperazinehexanamides: influence on lipophilicity and 5-HT7 receptor activity. Part III. J Med Chem.

[b28] Leopoldo M, Lacivita E, Berardi F, Perrone R, Hedlund PB (2011). Serotonin 5-HT7 receptor agents: structure-activity relationships and potential therapeutic applications in central nervous system disorders. Pharmacol Ther.

[b29] Lovell PJ, Bromidge SM, Dabbs S, Duckworth DM, Forbes IT, Jennings AJ (2000). A novel, potent, and selective 5-HT(7) antagonist: (R)-3-(2-(2-(4-methylpiperidin-1-yl)ethyl)pyrrolidine-1-sulfonyl) phenol (SB-269970). J Med Chem.

[b30] Martínez-García E, Leopoldo M, Lacivita E, Terrón JA (2011). Increase of capsaicin-induced trigeminal Fos-like immunoreactivity by 5-HT(7) receptors. Headache.

[b31] Matthys A, Haegeman G, Van Craenenbroeck K, Vanhoenacker P (2011). Role of the 5-HT7 receptor in the central nervous system: from current status to future perspectives. Mol Neurobiol.

[b32] Meltzer HY, Massey BW (2011). The role of serotonin receptors in the action of atypical antipsychotic drugs. Curr Opin Pharmacol.

[b33] Moore CA, Milano SK, Benovic JL (2007). Regulation of receptor trafficking by GRKs and arrestins. Annu Rev Physiol.

[b34] Perrone R, Berardi F, Colabufo NA, Lacivita E, Leopoldo M, Tortorella V (2003). Synthesis and structure-affinity relationships of 1-[omega-(4-aryl-1-piperazinyl)alkyl]-1-aryl ketones as 5-HT(7) receptor ligands. J Med Chem.

[b35] Pittalà V, Salerno L, Modica M, Siracusa MA, Romeo G (2007). 5-HT7 receptor ligands: recent developments and potential therapeutic applications. Mini Rev. Med. Chem.

[b36] Regard JB, Sato IT, Coughlin SR (2008). Anatomical profiling of G protein-coupled receptor expression. Cell.

[b37] Reynolds EE, Molinoff PB (1986). Down regulation of beta adrenergic receptors in S49 lymphoma cells induced by atypical agonists. J Pharmacol Exp Ther.

[b38] Smith C, Rahman T, Toohey N, Mazurkiewicz J, Herrick-Davis K, Teitler M (2006). Risperidone irreversibly binds to and inactivates the h5-HT7 serotonin receptor. Mol Pharmacol.

[b39] Sneddon WB, Magyar CE, Willick GE, Syme CA, Galbiati F, Bisello A (2004). Ligand-selective dissociation of activation and internalization of the parathyroid hormone (PTH) receptor: conditional efficacy of PTH peptide fragments. Endocrinology.

[b40] Stahl SM (2010). The serotonin-7 receptor as a novel therapeutic target. J Clin Psychiatry.

[b41] Thomas DR, Atkinson PJ, Hastie PG, Roberts JC, Middlemiss DN, Price GW (2000). [(3)H]-SB-269970–A selective antagonist radioligand for 5-HT(7) receptors. Br J Pharmacol.

[b42] Tokarski K, Zelek-Molik A, Duszynska B, Satała G, Bobula B, Kusek M (2012). Acute and repeated treatment with the 5-HT7 receptor antagonist SB 269970 induces functional desensitization of 5-HT7 receptors in rat hippocampus. Pharmacol. Rep.

[b43] Toohey N, Klein MT, Knight J, Smith C, Teitler M (2009). Human 5-HT7 receptor-induced inactivation of forskolin-stimulated adenylate cyclase by risperidone, 9-OH-risperidone and other “inactivating antagonists”. Mol Pharmacol.

[b44] Tsao P, von Zastrow M (2000). Downregulation of G protein-coupled receptors. Curr Opin Neurobiol.

[b45] Varin T, Gutiérrez-de-Terán H, Castro M, Brea J, Fabis F, Dauphin F (2010). Phe369(7.38) at human 5-HT(7) receptors confers interspecies selectivity to antagonists and partial agonists. Br J Pharmacol.

